# Group reminiscence for hope and resilience in care-seekers who have attempted suicide

**DOI:** 10.1186/s12991-020-0257-z

**Published:** 2020-01-15

**Authors:** Somayeh Hashemi-Aliabadi, Amir Jalali, Mahmoud Rahmati, Nader Salari

**Affiliations:** 1 Department of Psychiatric Nursing, School of Nursing and Midwifery, Kermanshah University of Medical Sciences, Kermanshah, Iran; 2 Substance Abuse Prevention Research Center, Research Institute for Health, Kermanshah University of Medical Sciences, Kermanshah, Iran; 3 Biostatistics Department, School of Nursing & Midwifery, Kermanshah University of Medical Sciences, Kermanshah, Iran

**Keywords:** Hope, Reminiscence, Resilience, Suicide

## Abstract

**Background:**

The rate of attempting suicide is growing due to the increasing social and economic problems and a variety of stresses taken by individuals in their lives. Helping people, boosting hope, and improving resilience to life hardships might be helpful in this area. This paper is an attempt to determine the effects of group reminiscence on hope and resilience in care-seekers who have attempted suicide.

**Method:**

The study was carried out as a quasi-experimental interventional study. The participants were 57 care-seekers with a history of attempting suicide who met the inclusion criteria. The sampling was done through convenience sampling and the participants were grouped into control (*n* = 29) and experimental (*n* = 28) groups randomly. The experimental group received integrated reminiscence sessions (eight sessions; 60–90 min). Hope and resilience of the subjects were measured using Schneider's Hope Scale and Connor and Davidson’s Resilience Scale. The scales were filled out by the subjects before, immediately after, and 4 weeks after the intervention.

**Results:**

The mean scores of hope in the experimental and control groups were 34.60 and 38.04, respectively, before the intervention. These figures immediately after the intervention were 44.07 and 35.96 in the experimental and control groups, respectively. 4 weeks after the intervention, the mean scores of hope in the experimental and control groups were 44.39 and 35.79, respectively, which is a statistically significant difference (*p* < 0.05). In terms of resilience, the mean scores in the experimental and control groups before the intervention were 48.17 and 57.51, respectively; and immediately after the intervention, these figures were 67.71 and 52.75, respectively. 4 weeks of the intervention, the mean scores of resilience were 59.17 and 52.24, respectively, which is a statistically significant difference (*p* < 0.05).

**Conclusion:**

Group reminiscence has a positive effect on boosting hope and resilience in care-seekers who have attempted suicide.

## Background

The recent years have witnessed an increase in suicide attempts due to an increase in life problems, a variety of stressors, and the pertinent thoughts and behaviors [1]. Committing suicide is a serious public health issue and an anti-social behavior [2]. It is an action with lethal outcomes that is intentionally attempted by oneself knowing about the deathly outcomes [3]. Suicide is the action of intentionally causing one’s death knowing the outcome of such an action [4].

Today, suicide is one of the main public health problems all around the world. Every year, about one million lose their lives by attempting suicide. It is the 13th cause of death in the world and the 3rd cause of death in the age group 15–44 years [5]. According to the World Health Organization (WHO), suicide rate in Iran was 4.1 out of 100 thousand before 2016 [6].

With regard to the pathology and the factors effective in suicide attempts, resilience is one of the main protective factors against suicide [7]. Suicide is undesirable resistive response to stress and hardships felt by the individual. Such reaction—i.e., attempting suicide—is directly affected by resilience [8]. One’s disability to face hardships and inefficient coping strategies or resilience results in inefficient coping with life crises and incidents [9]. Resilient individuals can overcome a variety of repercussions and preserve their mental health. That is, resilient individuals employ coping skills in a more efficient way [10].

The concept of hope is another predisposing factor and the same time a protective factor against attempting suicide [11]. Hope can play a key role in finding good solutions and planning and taking measure to solve problems. It is one of the factors in achieving better social successes [12]. Life without hope or with faint hope degrades one’s sense of self-efficacy and self-esteem so that they see no hope in trying for a better life. Consequently, those without hope tend to be less successful [11]. There is a close relationship between hope and optimism and hope is a trait commonly seen in the individuals searching for a better future. With hope deeply rooted in the mind and heart, the individual feels more passion for life [13]. Moreover, there is a negative relationship between hope and suicidal thoughts. Without hope, suicidal thoughts grow and self-care behaviors decrease [1].

Given the importance of suicide phenomenon, it is imperative to find efficient treatments based on scientific evidences. One of these scientific treatments for those who have attempted suicide is reminiscence [14]. Through reminiscence, individuals review their life as a natural interaction and general process, so that the individual reminisces their previous experiences, thinks about them, evaluates them, and ponders on them to achieve a deeper self-knowledge [15]. The reminiscence therapy is the basis of theoretical framework of Erikson’s theory of ego development [16]. In this method, the purposeful review and analysis of autobiographical memories are utilized as a core component of therapy [17]. Throughout reminiscence, individuals review their previous experiences, reframe them, reconstruct cognitive events of life, and expand their perception of the history of their personal life. These have a direct effect on emotions, feelings, behaviors, and cognition [18]. Evidences have shown that reminiscence-based therapy can be used as an effective way of controlling the symptoms of mental disorders [18] such as depression [14–16]. In addition, this method is a purposed approach to raise awareness and improve the quality of lives of individuals [19, 20]. Suicide is not a crime in Iran, but it is a socially abusive behavior and it is a sin according to the religious viewpoint.

Given the above introduction, it is essential to recognize effective treatments for care-seekers who have attempted suicide. The present paper is an attempt to determine the effectiveness of group reminiscence in hope and resilience in individuals who have attempted suicide.

## Materials and methods

The study was carried out as a quasi-experimental interventional study. Study population consisted of all care-seekers who had a history of attempting suicide visiting Imam Khomeni Hospital, Kermanshah-Iran. The participants were selected through convenience sampling and based on inclusion criteria. After being selected, the participants were grouped into control and experiment (sub-group A and B) groups as the intervention was done in two groups of 15 persons. There were men and women in the control and experiment (A, B) groups and women and men received therapeutic sessions separately.

**Fig. 1:**
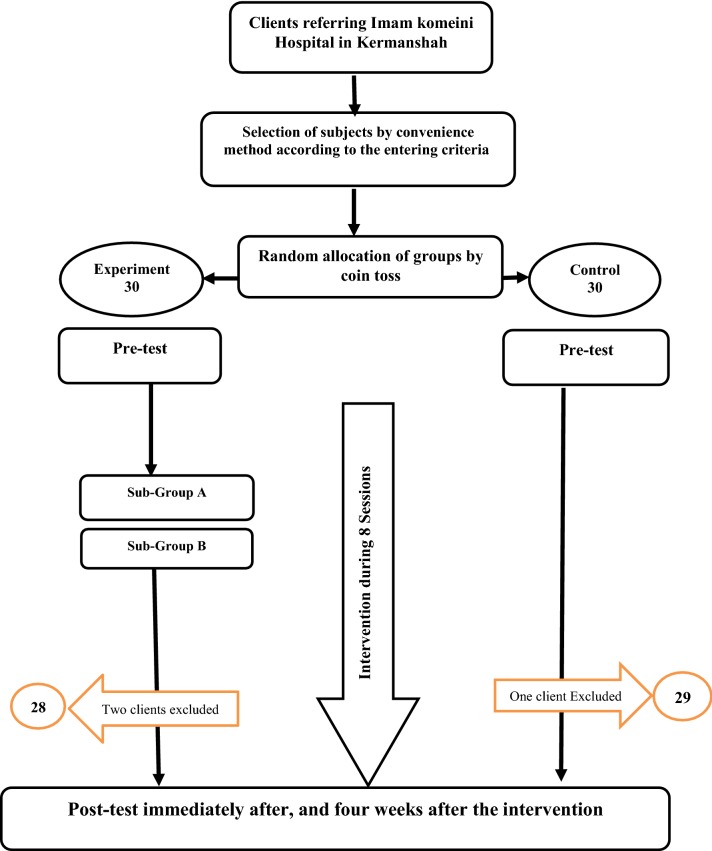
Diagram of the study steps

Inclusion criteria were age range 15–50, a good command of Farsi, no dependence on narcotic or psychedelic drugs, no acute psychosis and chronic/debilitating diseases, no cognitive diseases, and no recent similar treatments. Exclusion criteria were reluctance to continue participation in the study and absence in more than two sessions in the experiment group (Fig. 1).

### Sample size

The minimum number of participants was determined following Wu and Koo [20] for hope and Meléndez et al. [21] for resilience constructs and based on the formula for comparing one trait in two groups (confidence coefficient = 95% and test power = 90%).

### Data gathering tools

#### Demographics form

The form collects information like age, gender, marital status, education level, job, income level, and the number of children.

### Schneider's Hope Scale

The scale was introduced by Schneider et al. to measure hope with 12 self-statement questions. Agency thinking and pathway thinking each are covered by four questions and there are four distracting questions [22]. The minimum and maximum scores are 12 and 60, respectively, and the total score is obtained as the sum of the score of all questions. The higher the score, the higher the hope and vice versa. This scale is validated for Iranian population and the reported Cronbach’s alpha coefficients for the pathways and agency are 0.73 and 0.75, respectively [23].

### Connor and Davidson’s Resilience Scale

The scale contains 25 statements and designed to measure resilience in different individuals. The score is calculated as the sum of all statements and minimum and maximum scores are 0 and 100, respectively. The higher the score, the higher the resilience of the respondent and vice versa. The cut point of the scale is 50; that is, scores > 50 are interpreted as resilient and the higher the score the stronger the resilience and vice versa [24].

The tool was normalized by Ghanei-Gheshlagh et al. [25] for patients with cardiovascular and respiratory diseases. Cronbach’s alpha coefficient of the final version of the scale is 0.943.

### Study process

The study was started after approval of the research plan by Higher Education Committee (No.: 96298) and Ethics Committee of Kermanshah University of Medical Sciences (KUMS.REC.1396.258). After making the required arrangements with the university officials, the authors visited Imam Khomeni Hospital and won the support of hospital officials by briefing them about the objectives. Sampling process was done with consent of the candidates and based on inclusion criteria. Totally, 60 participants were selected and randomly grouped into experiment (sub-group A & B) and control groups each with 30 members. The participants signed a written consent form before filling out the demographics form and Hope and Resilience scales. The participants in the experiment group were divided into two sub-groups (A & B, *n* = 15) and took part in eight integrated reminiscence session (twice a week; 1.5 h each). Immediately after and 4 weeks after the intervention, the participants of the two groups filled out the two scales. Throughout the study two subjects in the experiment group (sub-group B) were excluded at 6th and 8th weeks due to absence and one subject in the control group was excluded due to failure to fill out the questionnaire four weeks after the interventions. Therefore, the study was continued with 57 participants (Fig. 1).

The intervention package was designed based in library studies and literature review. It was provided to 10 faculty board members and researchers in the field and the feedbacks were used for modification of the package (Table 1).

**Table 1:** Topics covered in the integrated reminiscence sessions

Session	Title	Objective	Techniques
1	Introduction Objectives and regulations Achieving a single approach Explaining the reminiscence sessions and the outcomes	Introducing the participants to each other Creating a casual environment Attracting attention and interest Introducing the objectives Introducing the process of reminiscence	Warm up Communicational skills Giving identification Clarification
2	Sharing memories about oppressed feeling, loneliness, and hopelessness	Emotional ventilation Recognizing feelings and positive/negative emotions	Free association Re-expression Reflecting Socratic questioning
3	Sharing memories about anxiety, anger, and fear	Emotional ventilation, altering cognition in the participant, and anger and anxiety management	Free association Re-expression Clarification Socratic questioning
4	Sharing memories about the first experience with affection and love, optimism and negativism, fate and free will	Emotional ventilation Improving self-awareness Altering one’s will	Free association Re-expression Clarification Socratic questioning
5	Sharing memories about physical violence and feelings in childhood and at school	Emotional ventilation Recognizing individuals’ mood Eliminating unrelaxing feelings	Free association Re-expression Clarification Purposeful questioning
6	Sharing memories about body mental image, subjective mental image and life mental image	Recognizing the pain of inferiority complex Recovering and modifying mental images	Free association Re-expression Clarification Socratic questioning
7	Sharing memories about positive and moral feelings and negative destructive feelings	Recognizing weaknesses and strengths Familiarizing with bothering feelings and eliminating negative feelings	Free association Re-expression Clarification Socratic questioning
8	Summarizing and celebrating Hearing comments about the meanings of sessions	Surveying participants’ thoughts and feelings about group work Planning for future Thanking the participants	Summarizing Developing concept

All the sessions were featured with discussing about commonalities and differences between memories and summarizing the main aspects of the subject in hand. At the final session, the participants shared their feelings and thoughts about the group experience, the closing event, the main aspects of the session and planning for the future. The leader also gave a brief review of the previous sessions. To renew the positive mental memories, the leader expressed his feeling about the conclusion of sessions and the participants were also asked to do so. Eventually, the participants were asked to share their plans for the near future with regard to their experiences in the group [23].

## Results

Totally, 57 individuals with a history of attempting suicide took part in the study in two groups of experiment (*n* = 28) and control (*n* = 29). Table 2 lists the demographics of the participants. As listed, 27 men and 30 women participated. Mean age of the participants was 26.38 years. The mean age of the subjects in the experiment and control groups were 26.73 ± 8.23 and 25.32 ± 7.94, respectively, there was no significant difference between the two groups in terms of the mean age. In addition, based on the Kolmogorov Smirnoff, the distribution of age variable was normal in the both groups. Demographical variables were compared between the two groups using Chi-squared test and the results showed no significant difference between the two groups in terms of demographics (Table 2).

**Table 2:** Frequency comparison of demographic characters of suicide attempters in experimental and control groups

Variables	Control		Experiment		Sum		Chi^2^	*df*	*p *value
	No	%	No	%	No	%			
Gender									
Male	15	51.7	12	42.9	27	47.4	0.449	1	0.503
Female	14	48.3	16	57.1	30	52.6			
Marital status									
Single	19	65.5	15	53.6	34	59.6	0845	1	0.358
Married	10	34.5	13	46.4	23	40.4			
Graduate level									
High School	19	65.5	20	71.4	39	68.4	0.23	1	0.631
Higher Ed	10	34.5	8	28.6	18	1.6			
Job									
Employed	7	24.1	8	28.6	15	26.3	0.217	3	0.975
Unemployed	6	20.7	5	17.9	11	19.3			
House wife	9	31	9	32.1	18	31.6			
Student	7	24.1	6	21.4	13	22.8			
Income (month)									
< 500$	26	89.7	23	82.1	49	86	0.666	1	0.414
> 500$	3	10.3	5	17.9	8	14			
Have a child									
No	24	82.8	21	75	45	78.9	0.516	1	0.473
Yes	5	17.2	7	25	12	21.1			
History of suicide									
Once	22	75.9	22	78.6	44	77.2	0.059	1	0.807
> More than once	7	24.1	6	21.4	13	22.8			
Last time of suicide									
A month ego	7	24.1	4	14.3	11	19.3	1.087	3	0.78
Two months ego	10	34.5	10	35.7	20	35.1			
Three months ego	6	20.7	6	21.4	12	21.1			
Four months ego	6	20.7	8	28.6	14	24.6			

Normal distribution of the quantitative demographical data including age and hope and resilience scores before, immediately after, and 4 weeks after the intervention in the two groups was tested using Kolmogorov–Smirnov (KS) and Shapiro tests. As the tests showed, all variables had a normal distribution and only resilience in the control group was not normally distributed at the three measurement occasions.

The results showed that mean age of the control and experiment groups was 26.72 and 25.32, respectively and independent *t*-test showed no significant difference between two groups so that the two groups were homogeneous in this regard (*p* = 0.516).

The mean score of hope in the experiment and control groups before the intervention was 35.89 and 37.66, respectively; these figures were 42.1 and 37.46, respectively, immediately after the intervention. Four weeks after the intervention, the mean score of hope in the experiment and control groups was 42.25 and 37.28, respectively, and based on ANOVA test, the growing trend in the intervention group was significant (*p* = 0.0001). In addition, based on post hoc Tukey test, the growing trend between before the intervention and immediately afterwards was significant (*p* = 0.04) and insignificant between immediately and four weeks after the intervention (*p* = 0.87). On the other hand, the change trend in the control group was insignificant (*p* = 0.386) and independent t-test showed no significant difference between the control and experiment groups before the intervention (*p* = 0.15). This means that the two groups were homogeneous before the intervention in terms of hope. However, there was a significant difference between the two groups in terms of hope immediately (*p* = 0.15) and four weeks (*p* = 0.001) after the intervention (Table 3).

**Table 3:** Comparison of mean score of hope in three positions "before, after, and Four weeks after" in suicide attempters in control and Experimental groups

Group	Time			
	Before	After	Four weeks later	
Control	37.66 ± 7.61	37.46 ± 7.58	37.28 ± 7.51	*F* = 0.968 *p* = 0.386
Experiment	35.89 ± 816	42.1 ± 6.36	42.25 ± 6.44	*F* = 7.5 *p* = 0.001
	*T* = − 0.842 *p* = 0.403	*T* = 2.5 *p* = 0.015	*T* = 2.68 *p* = 0.01	

The mean score of resilience in the experiment and control groups before the intervention was 51.54 and 51.24, respectively; these figures were 60.93 and 51.86, respectively, immediately after the intervention. Four weeks after the intervention, the mean score of resilience in the experiment and control groups was 61.78 and 51.38, respectively, and based on repeated measures ANOVA test, the growing trend in the experiment group was significant (*p* = 0.008). In addition, based on post hoc Tukey test, the growing trend between before the intervention and immediately afterwards was significant (*p* = 0.017) and insignificant between immediately and four weeks after the intervention (*p* = 0.96). On the other hand, the change trend in the control group was insignificant (*p* = 0.272) and Mann Whitney test showed no significant difference between the control and intervention groups before the intervention (*p* = 0.987). This means that the two groups were homogeneous before the intervention in terms of resilience. However, there was a significant difference between the two groups in terms of resilience immediately (*p* = 0.027) and four weeks (*p* = 0.018) after the intervention (Table 4).

**Table 4:** Comparison of mean score of resilience in three positions "before, after, and Four weeks after" in suicide attempters in control and experimental groups

Group	Time			
	Before	After	Four weeks after	
Control	51.24 ± 13.14	51.86 ± 13.6	51.38 ± 13.48	*Z* = 1.37 *p* = 0.272
Experiment	51.54 ± 14.28	60.93 ± 285	61.78 ± 13.01	*F* = 3.86 *p* = 0.008
	*Z* = − 0.016 *p* = 0.987	*Z* = − 2.21 *p* = 0.027	*Z* = − 2.37 *p* = 0.018	

## Discussion

Group reminiscence was effective in hope in the experiment group, while there was no change in the control group in terms of hope. Chiang found that reminiscence led to an improvement in hope and loneliness feeling [26]. In a study, Wu argued that self-reminiscence was a tool to achieve integrity and assigning meaning to memories and life trend, which led to higher hope [20]. In addition, Mackinlay and Trevitt [19] reported that reminiscence was a way to give meaning to positive and negative experiences of life.

Group reminiscence therapy increased resilience score in the experiment group. This finding is consistent with other studies [17, 27, 28]. To explain this, reminiscence helps people to desert the thinking and behavior styles that have degraded their self-esteem and social relationships over time while it helps them to alleviate their negative feelings about themselves and others [29]. In addition, reminiscing and reviewing the life are effective in boosting individuals’ self-confidence so that people create a positive feeling of their identity by sharing memories. This practice has a valuable role in solving unsolved life issues and negative memories. Throughout reminiscing, people re-evaluate their negative and positive experiences through reconstructing their life event and this leads to a lower depression and higher resilience [30]. Gaggioli et al. [27] found that reminiscence had a significant effect on decreasing the symptoms of depression and suicidal thoughts, while it improved resilience in subjects. Melendez-Moral et al. (2013) argued that reminiscence resulted in a significant improvement in self-esteem, resilience, and interpersonal relationship in care-seekers [28]. Hallford and Mellor [17] reported that reminiscence created a significant improvement in adjusting emotions, resilience, and quality of sleep in the subjects. To explain the findings, it is notable that resilience can function as an intermediating mechanism through improving self-esteem and lead to positive adaptability. In addition, with low resilience, one loses self-esteem and the process of fighting negative experience loses its efficiency [31]. Moreover, group approach of the intervention can be helpful in developing the idea in the participants that damages, shortages, problems, and obstacles are normal and inevitable things in life. In addition, the group nature of intervention makes it highly effective in improving resilience through decreasing catastrophic thinking about events [32].

As to the limitations of the study, the problems in sampling process are notable. Sampling and intervention process took 11 months. Many care-seekers were not interested in participation and the authors had to hold several meetings with the candidates to ensure them about the objectives and confidentiality of their information and convince them to participate in the study. The participants were selected based on their medical history and information available in their case files. Any possible health problems or treatments that were not mentioned in the medical files or not stated in the file were not included. This can be considered as a limitation of the study. Time was another limitation, so that the follow-up term was limited to 4 weeks. With 3 months follow-up term, the results would have been more reliability.

## Conclusion

Group reminiscence is a reliable and proper approach for improving resilience and hope in care-seekers with a history of suicide attempt. An improvement in resilience and hope following reminiscence session is expected. Such interventions are opportunities for emotional ventilation, cognitional alteration, improving self-awareness, changing the participant’s will, and recovering and correcting mental images.

## Data Availability

The datasets used and analyzed during the current study are available from the corresponding author on reasonable request.

## Abbreviations

KS: Kolmogorov–Smirnov
KUMS: Kermanshah University of Medical Sciences

## References

[1] Huen JMY, Ip BYT, Ho SMY, Yip PSF (2015). Hope and Hopelessness: The Role of Hope in Buffering the Impact of Hopelessness on Suicidal Ideation. PLoS ONE.

[2] Bjorkenstam C, Ekselius L, Berlin M, Gerdin B, Bjorkenstam E (2016). Suicide risk and suicide method in patients with personality disorders. J Psychiatr Res.

[3] Sadock BJ, Sadock VA, MD PR. Kaplan and Sadock's synopsis of psychiatry: behavioral science clinical psychiatry, 11th edition. Philadelphia: Wolters Kluwer; 2014, p. 897–906.

[4] Carlén P, Bengtsson A (2007). Suicidal patients as experienced by psychiatric nurses in inpatient care. Int J Mental Health Nurs..

[5] Rogers ML, Joiner TE (2018). Suicide-specific rumination relates to lifetime suicide attempts above and beyond a variety of other suicide risk factors. J Psychiatr Res.

[6] WHO (2018). World health statistics 2018: monitoring health for the SDGs, sustainable development goals.

[7] Heisel MJ, Flett GL (2016). Does recognition of meaning in life confer resiliency to suicide ideation among community-residing older adults? A longitudinal investigation. Am J Geriatr Psychiatry..

[8] Wood L, Ntaote GM, Theron L (2012). Supporting Lesotho teachers to develop resilience in the face of the HIV and AIDS pandemic. Teach Teach Educ..

[9] Boyle G, Saklofske D, Matthews G (2015). Measures of Personality and Social Psychological Constructs.

[10] Davydov DM, Stewart R, Ritchie K, Chaudieu I (2010). Resilience and mental health. Clin Psychol Rev..

[11] Luo X, Wang Q, Wang X, Cai T (2016). Reasons for living and hope as the protective factors against suicidality in Chinese patients with depression: a cross sectional study. BMC Psychiatry..

[12] Mohammadi F, Fard FD, Heidari H (2014). Effectiveness of Logo Therapy in Hope of Life in the Women Depression. Procedia Soc Behav Sci..

[13] Tarhan S, Bacanlı H, Dombaycı MA, Demir M (2011). Quadruple thinking: hopeful thinking. Procedia Soc Behavioral Sci..

[14] Song D, Shen Q, Xu T-Z, Sun Q-H (2014). Effects of group reminiscence on elderly depression: A meta-analysis. Int J Nurs Sci..

[15] Elias SM, Neville C, Scott T (2015). The effectiveness of group reminiscence therapy for loneliness, anxiety and depression in older adults in long-term care: a systematic review. Geriatr Nurs..

[16] Chen TJ, Li HJ, Li J (2012). The effects of reminiscence therapy on depressive symptoms of Chinese elderly: study protocol of a randomized controlled trial. BMC Psychiatry..

[17] Hallford DJ, Mellor D (2013). Cognitive-reminiscence therapy and usual care for depression in young adults: study protocol for a randomized controlled trial. Trials..

[18] Musavi M, Mohammadian S, Mohammadinezhad B (2017). The effect of group integrative reminiscence therapy on mental health among older women living in Iranian nursing homes. Nurs Open..

[19] Mackinlay E, Trevitt C (2010). Living in aged care: using spiritual reminiscence to enhance meaning in life for those with dementia. Int J Mental Health Nurs..

[20] Wu LF, Koo M (2016). Randomized controlled trial of a six-week spiritual reminiscence intervention on hope, life satisfaction, and spiritual well-being in elderly with mild and moderate dementia. Int J Geriatr Psychiatry..

[21] Melendez JC, Fortuna FB, Sales A, Mayordomo T (2015). The effects of instrumental reminiscence on resilience and coping in elderly. Arch Gerontol Geriatr.

[22] Schneider MC, Castillo-Salgado C, Bacallao J, Loyola E, Mujica OJ, Vidaurre M (2005). Summary of indicators most used for the measurement of the health inequalities. Epidemiol Bull.

[23] Shegefti NS, Samani S (2011). Psychometric properties of the academic hope scale: Persian form. Procedia Soc Behav Sci..

[24] Connor KM, Davidson JR (2003). Development of new resilience scale: the Connor- Davidson, Resilience scale (CD-RISC). Depress Anxiety..

[25] Gheshlagh RG, Tabrizi KN, Dalvandi A, Ebadi A (2018). Development and validation of resilience scale in patients with cardiovascular and respiratory diseases. Iranian Red Crescent Med J..

[26] Chiang KJ, Chu H, Chang HJ, Chung MH, Chen CH, Chiou HY (2010). The effects of reminiscence therapy on psychological well-being, depression, and loneliness among the institutionalized aged. Int J Geriatric Psychiatry..

[27] Gaggioli A, Scaratti C, Morganti L, Stramba-Badiale M, Agostoni M, Spatola CA (2014). Effectiveness of group reminiscence for improving wellbeing of institutionalized elderly adults: study protocol for a randomized controlled trial. Trials..

[28] Melendez-Moral JC, Charco-Ruiz L, Mayordomo-Rodriguez T, Sales-Galan A (2013). Effects of a reminiscence program among institutionalized elderly adults. Psicothema..

[29] Woods B, Aguirre E, Spector AE, Orrell M (2012). Cognitive stimulation to improve cognitive functioning in people with dementia. Cochrane Database Syst Rev.

[30] Stinson CK, Kirk E (2006). Structured reminiscence: an intervention to decrease depression and increase self-transcendence in older women. J Clin Nurs.

[31] Bang JS, Jo S, Kim GB, Kwon BS, Bae EJ, Noh CI (2013). The mental health and quality of life of adult patients with congenital heart disease. Int J Cardiol..

[32] Van Bogaert P, Tolson D, Eerlingen R, Carvers D, Wouters K, Paque K (2016). SolCos model-based individual reminiscence for older adults with mild to moderate dementia in nursing homes: a randomized controlled intervention study. J Psychiatr Mental Health Nurs..

